# Association of Impaired Hematoma Clearance After Aneurysmal Subarachnoid Hemorrhage with Delayed Cerebral Ischemia, Hydrocephalus, and Long-Term Outcomes

**DOI:** 10.1007/s12028-026-02525-z

**Published:** 2026-04-23

**Authors:** Kokyo Sakurada, Takuma Maeda, Yushiro Take, Natsuki Shozuhara, Jun Yanagida, Kuya Azekami, Hiroki Kobayashi, Akio Teranishi, Tomomichi Kayahara, Hiroki Kurita

**Affiliations:** Department of Cerebrovascular Surgery, Saitama Medical University International Medical Center, Hidaka, Japan

**Keywords:** Subarachnoid hemorrhage, Intracranial aneurysm, Delayed cerebral ischemia, Hydrocephalus, Treatment outcome, Hematoma clearance

## Abstract

**Objective:**

Although delayed cerebral ischemia (DCI) and hydrocephalus contribute to poor outcomes after aneurysmal subarachnoid hemorrhage (aSAH), it remains unclear whether improved subarachnoid hematoma clearance translates into better long-term outcomes and through which mechanisms. We investigated the association between hematoma clearance and long-term outcomes and quantified the mediating effects of DCI and shunt-dependent hydrocephalus.

**Methods:**

We retrospectively analyzed patients with aSAH who underwent aneurysmal clipping between April 2012 and March 2024 at a single center. Hematoma burden was assessed using the Hijdra sum score (HSS). Hematoma clearance was defined as the percentage reduction in HSS between admission and days 7‒10 and dichotomized using the median (91.5%). Poor outcome was defined as a modified Rankin Scale (mRS) score ≥ 3 at 6 months. Multivariate logistic regression analyses and causal mediation analyses were performed to assess direct and indirect effects of hematoma clearance on long-term outcomes mediated by DCI and shunt-dependent hydrocephalus.

**Results:**

A total of 442 patients were included. Poor hematoma clearance was independently associated with poor 6-month outcomes [odds ratio (OR) 2.32, *p* = 0.003], DCI (OR 1.83, *p* = 0.025), and shunt-dependent hydrocephalus (OR 2.55, *p* < 0.001). Mediation analysis showed that shunt-dependent hydrocephalus mediated approximately 22% of the total effect of poor clearance on poor outcome [average causal mediation effect (ACME) 0.035, *p* < 0.001], whereas mediation via DCI was smaller and did not reach statistical significance (ACME 0.020 *p* = 0.078). A substantial direct effect independent of these mediators was observed.

**Conclusions:**

Poor hematoma clearance after aSAH is associated with worse long-term outcomes, partly mediated by shunt-dependent hydrocephalus and to a lesser extent by DCI. However, a large proportion of the effect appears to be independent of these pathways, suggesting the involvement of additional mechanisms beyond DCI and hydrocephalus.

**Supplementary Information:**

The online version contains supplementary material available at https://doi.org/10.1007/s12028-026-02525-z.

## Introduction

Long-term outcomes of aneurysmal subarachnoid hemorrhage (aSAH) are adversely affected by mediating pathologies, including delayed cerebral ischemia (DCI) and hydrocephalus [1]. These mediating pathologies are thought to develop, at least in part, because of degradation products derived from residual subarachnoid blood [2].

Based on this concept, there are many interventions aiming at facilitating the clearance of subarachnoid hematoma for reducing the risk of DCI and hydrocephalus by removing the degradation products [3–7]. Several studies have demonstrated an association between the initial hematoma burden and secondary complications, including DCI, hydrocephalus, and early brain injury (EBI), as well as long-term outcomes [8–11]. However, evidence directly linking the rate of hematoma clearance to long-term neurological outcomes remains limited. Furthermore, only a small number of studies have reported the association between clearance and the mediating pathologies such as DCI and hydrocephalus [12–14]. As a result, it remains unclear to what extent efforts to enhance hematoma clearance contribute to improved long-term outcomes through the reduction of DCI and hydrocephalus.

Therefore, this study investigated whether subarachnoid hematoma clearance influences the long-term outcome of aSAH and quantified the extent to which this association is mediated by DCI and shunt-dependent hydrocephalus.

## Methods

### Patient Selection

This single-center retrospective study included patients with aSAH who underwent aneurysmal clipping at Saitama Medical University International Medical Center between April 2012 and March 2024. This study was reviewed and approved by Institutional Review Board of Saitama Medical University International Medical Center (2025-086) and did not require written informed consent owing to its opt-out design. Participants were informed of the study and could decline.

At our institution, surgical clipping was generally indicated for patients with aSAH presenting within 72 h of hemorrhage. However, clipping was not performed in patients who exhibited extensive established cerebral infarction or loss of brainstem reflexes due to cerebral herniation. The selection of treatment modality was determined by consensus between the attending cerebrovascular surgeons and endovascular specialists.

The inclusion criteria were as follows: (1) age ≥ 18 years with a diagnosis of SAH confirmed by computed tomography (CT); (2) presence of an intracranial aneurysm identified by digital subtraction angiography (DSA), CT angiography, or magnetic resonance imaging (MRI), and confirmed intraoperatively; and (3) surgical clipping performed within 72 h after aneurysmal rupture.

The exclusion criteria were as follows: (1) extensive ischemic lesion detected immediately after surgery, defined as infarction involving more than one vascular territory or causing significant mass effect. These patients were excluded because the presence of extensive postoperative infarction makes it difficult to distinguish new neurological deterioration due to DCI or hydrocephalus from deficits attributable to the infarction itself; (2) death within 7 days after hemorrhage; (3) absence of CT imaging obtained between days 7 and 10 after hemorrhage; (4) unavailability of the modified Rankin Scale (mRS) score at 6 months; and (5) rebleeding after surgical clipping.

### Surgical Strategy of Clipping

During surgical clipping, irrigation of the subarachnoid space was performed according to the surgical approach. In the transsylvian approach, the ipsilateral sylvian fissure and basal cistern were irrigated. In the interhemispheric approach, the interhemispheric and basal cisterns were irrigated. In other approaches, the relevant cisterns were irrigated as appropriate.

### Management of SAH

The patients were admitted to the intensive care unit after surgical clipping and monitored up to day 14 after onset. Based on our stroke team’s protocol, patients underwent a CT scan at least once on postoperative day 1 and again between days 7 and 10 after stroke onset. MRI screening for DCI was routinely performed on days 7 and 14. Additionally, CT or MRI was performed whenever patients exhibited new symptoms of neurological deficit. Patients were administered 5% albumin and red blood cells as required to maintain serum albumin more than 2.5 g/dL and hemoglobin (Hb) higher than 8 g/dL. To prevent vasospasm, cilostazol, ozagrel, and fasudil were routinely administered from days 4 to 14. Additionally, after June 2022, clazosentan was included in the treatment protocol.

### Cisternal Lavage

Intraoperatively, ventricular and cisternal drains were placed. During surgical clipping, an external ventricular drain was placed in the anterior horn of the lateral ventricle for anterior circulation aneurysms, and in the posterior horn for posterior circulation aneurysms. Additionally, a cisternal drain was placed in the opticocarotid cistern during the pterional approach and in the prechiasmatic cistern during the interhemispheric approach. For posterior circulation aneurysms, a lumbar spinal drain was inserted instead of a cisternal drain. After confirming that no bleeding had occurred, irrigation at a flow rate of 20 mL/h was initiated. The cisternal irrigation solute was a mixture of 500 mL of lactic acid Ringer’s lactate solution, 2000 mg of ascorbic acid, and 4 mEq of magnesium sulfate. We used magnesium sulfate in the irrigation solute, which acts as a calcium antagonist [15]. Irrigation was discontinued in the case of acute brain swelling, rebleeding, or hydrocephalus. The cisternal drainage tube was connected to the pressure-control system via a T-connector as a safety outlet. The overflow route was set at 25 cm H_2_O to prevent excessive infusion. Cisternal or spinal drainage was started 15 cm above the level of the external auditory meatus and adjusted to a height that allowed 200 mL of cerebrospinal fluid (CSF) to be drained per day. All drainage catheters were carefully monitored to prevent complications such as over-drainage and infection. All drainages were gradually weaned once the risk of cerebral vasospasm was considered to have subsided on the basis of clinical and radiological assessment. The drain height was increased to 20 cm H_2_O, followed by CT imaging to confirm the absence of ventricular enlargement prior to drainage removal. Although cisternal lavage was routinely performed in our institution, some patients had only a single drain placed because of brain swelling or other intraoperative considerations. For analysis, patients were considered to have undergone cisternal lavage if both ventricular and cisternal (or spinal) drains were placed. Patients with only a single drain were classified as not having received cisternal lavage.

### Management of DCI

When DCI was suspected on the basis of new focal neurological deficits or a decrease in consciousness not explained by other causes, urgent CT or MRI was performed to exclude other pathology. Hemodynamic augmentation therapy was initiated, aiming to maintain systolic blood pressure above 140 mmHg. In cases refractory to medical therapy, endovascular rescue treatment, including intra-arterial infusion of fasudil hydrochloride or balloon angioplasty, was considered.

### Data Collection and Definitions

We collected the following demographic data from the patients’ clinical records: age, sex, smoking history, hypertension (HT), and diabetes mellitus (DM). The severity of the neurological condition at adminission was evaluated using the World Federation of Neurological Surgeons (WFNS) grading system. We evaluated the following imaging characteristics: modified Fisher scale (mFisher) [16], Hijdra sum score (HSS) [17], location of aneurysms, and maximum diameter of the aneurysms. We evaluated HSS on CT scans at admission and on days 7–10. The HSS reduction rate was defined as the hematoma clearance parameter.

$$\text{HSS reduction rate}_{\mathrm{Days} \,7-10} (\%) = \frac{\mathrm{HSS}_{\mathrm{preoperative}} - \mathrm{HSS}_{\mathrm{Days} \,7-10}}{\mathrm{HSS}_{\mathrm{preoperative}}} \times 100$$

We categorized the HSS reduction rate_Days 7–10_ on the basis of its median value of 91.5%. We defined HSS reduction rate_Days 7–10_ < 91.5% as “poor clearance.”

### Primary and Secondary Outcomes

The primary outcome was functional outcome at 6 months after onset, with poor outcome defined as an mRS score ≥ 3.

The secondary outcomes included the occurrence of DCI and shunt-dependent hydrocephalus.

DCI was defined as the presence of new focal neurological deficits observed between 4 and 14 days after onset or a decrease of 2 points or more on the Glasgow Coma Scale [18]. Additionally, in patients under sedation or with severely impaired consciousness, in whom focal neurological deficits could not be assessed, new infarct findings in the vascular territory or watershed area identified by MRI or CT were included in the diagnosis of DCI. Shunt-dependent hydrocephalus was defined as secondary hydrocephalus occurring later than 14 days after onset and requiring a ventriculoperitoneal shunt or lumboperitoneal shunt. Because clazosentan was introduced after June 2022, we compared the difference of the primary and the secondary outcomes between the patients who were and were not administered clazosentan.

### Causal Mediation Analysis

To quantify the mediating effects of DCI and shunt-dependent hydrocephalus on the relationship between the HSS reduction rate and mRS at 6 months, a causal mediation analysis was performed.

### Robustness Analysis

To assess the robustness of our findings, we varied the definition of poor clearance using 25th percentile (83.3%) and 75th percentile (100.0%) in the multivariate and causal mediation analyses.

### Statistical Analysis

All statistical analyses were performed using R version 4.5.0. Continuous variables were compared using Welch’s two-sample *t*-test or the Mann–Whitney *U* test, as appropriate. Categorical variables were compared using Pearson’s chi-squared test.

### Multivariate Analysis

Univariate analyses were conducted to identify potential covariates associated with clinical outcomes. The factors included age, sex, smoking history, HT and DM history, mFisher scale score of 3–4, WFNS grade of IV–V, maximum dome size, HSS on admission, and poor clearance [19–22]. Variables demonstrating a significance level of *p* ≤ 0.10 in the univariate analyses were subsequently included in the multivariate logistic regression model.

In the causal mediation analysis, the R package “mediation” (version 4.5.0) was used. First, two logistic regression models were constructed: one mediator model predicting DCI and shunt-dependent hydrocephalus as mediators, and an outcome model including these mediators to predict poor mRS. Variables consistently incorporated in multivariate logistic regression analyses for DCI, shunt-dependent hydrocephalus, and poor mRS were included in the mediation models.

A causal mediation analysis was used to estimate the average causal mediation effect (ACME), average direct effect (ADE), and total effect. These estimates were obtained using 1000 iterations of nonparametric bootstrapping, with significance evaluated on the basis of 95% confidence intervals (CIs) calculated using the percentile method.

### Statistical Significance

All tests were two-tailed, and *p* < 0.05 was considered statistically significant. Additionally, *p* < 0.001 was interpreted as providing stronger evidence against the null hypothesis.

## Results

**Fig. 1:**
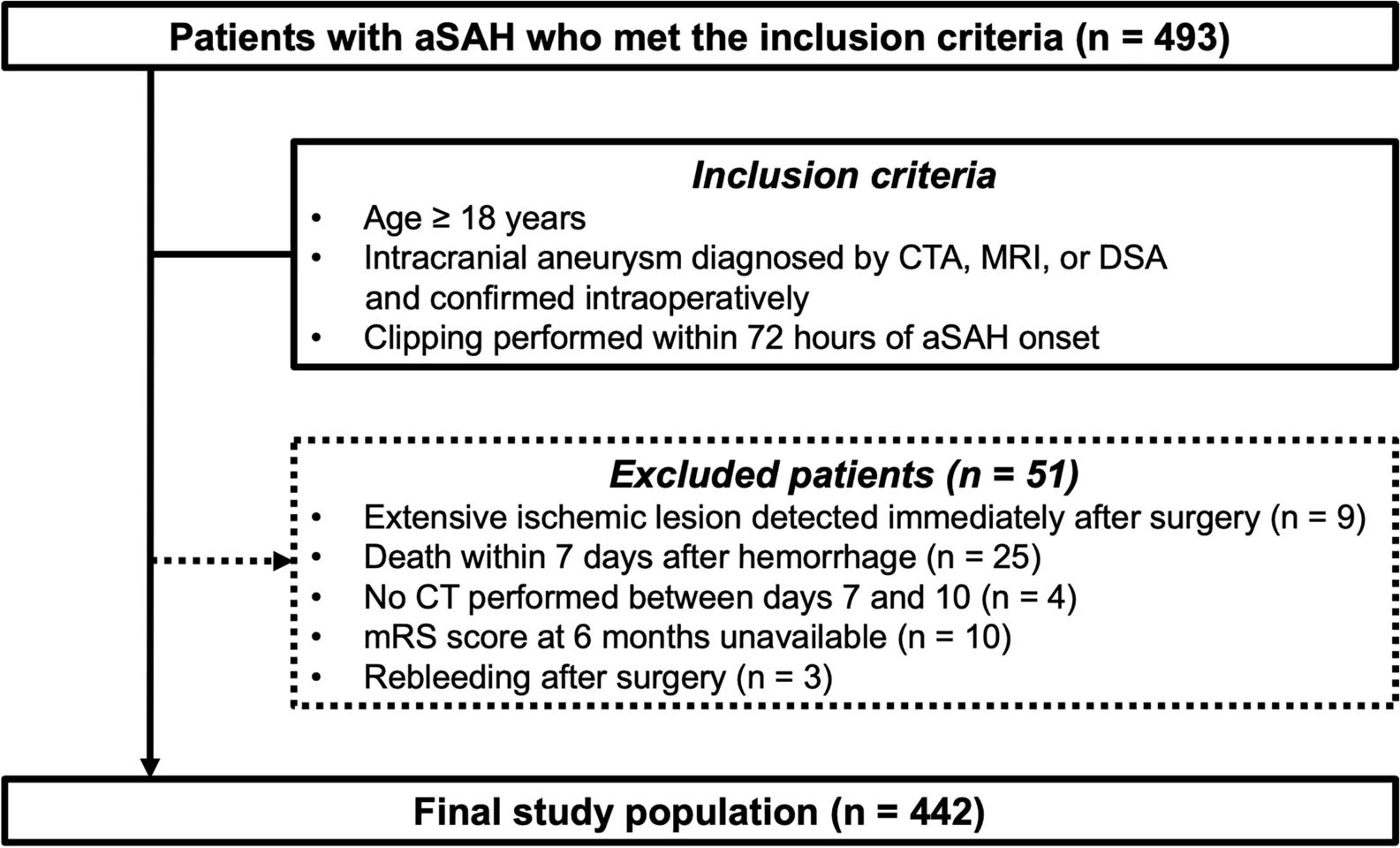
Flow diagram of study patients. aSAH, aneurysmal subarachnoid hemorrhage; CT, computed tomography; CTA, computed tomographic angiography; DSA, digital subtraction angiography; MRI, magnetic resonance imaging; mRS, modified Rankin Scale

A total of 493 patients were included in the study. Of these, nine patients were excluded owing to the detection of an extensive ischemic lesion immediately after surgery. In addition, 25 patients were excluded owing to death within 7 days. Four patients were excluded owing to the absence of a CT scan between 7 and 10 days after hemorrhage. In addition, ten patients were excluded owing to an unavailable mRS score at 6 months, and three patients were excluded owing to rebleeding. Finally, data from 442 patients were analyzed (Fig. 1).

The poor clearance group had a higher mean age (66 vs. 60 years, *p* < 0.001), a higher proportion of WFNS IV–V (51% vs. 32%, *p* < 0.001) and mFisher scale 3–4 (86% vs. 75%, *p* = 0.003), and a higher median HSS (24 vs. 20 points, *p* = 0.002) (Table 1). Additionally, the proportions of DCI, shunt-dependent hydrocephalus, and poor mRS were significantly higher in the poor clearance group (DCI: 27% vs. 13%, *p* < 0.001; shunt-dependent hydrocephalus: 35% vs. 14%, *p* < 0.001; poor mRS: 53% vs. 35%, *p* < 0.001) (Table 1).

**Table 1:** Summary table of included patients divided into two groups based onon the basis of the hematoma clearance

	Hematoma Clearanceclearance			
	Total	Good *N* = 216	Poor *N* = 226	*P*-value
Age, mean (SD), years	63 ± 14	60 ± 15	66 ± 13	< 0.001
Sex, Female, *n* (%)	286 (65%)	138 (64%)	148 (65%)	0.725
Smoking, *n* (%)	127 (35%)	65 (35%)	62 (35%)	0.921
HT, *n* (%)	260 (59%)	131 (61%)	129 (58%)	0.511
DM, *n* (%)	36 (9%)	14 (7%)	22 (10%)	0.293
WFNS grade of IV–V, *n* (%)	186 (42%)	70 (32%)	116 (51%)	< 0.001
Modified Fisher group of 3–4, *n* (%)	355 (80%)	161 (75%)	194 (86%)	0.003
HSS on admission, median (P25–P75)	21 (15, 27)	20 (13, 26)	24 (15, 29)	0.002
HSS Reduction reduction Rrate _*Days 7-10*_, mean (SD)	87.7 ± 15.6	98.0 ± 2.8	77.8 ± 16.4	< 0.001
Location of ruptured aneurysm, *n* (%)				0.700
ACA	153 (35%)	73 (34%)	80 (35%)	
ICA	128 (29%)	66 (31%)	62 (27%)	
MCA	147 (33%)	72 (33%)	75 (33%)	
Posterior circulation	14 (3%)	5 (2%)	9 (4%)	
Aneurysm size, mean (SD)	6.0 ± 3.2	6.2 ± 3.4	5.9 ± 2.9	0.324
Cisternal lavage, *n* (%)	365 (83%)	182 (84%)	183 (81%)	0.416
DCI, *n* (%)	90 (20%)	28 (13%)	62 (27%)	< 0.001
Shunt-dependent hydrocephalus, *n* (%)	109 (25%)	30 (14%)	79 (35%)	< 0.001
Poor mRS, *n* (%)	171 (39%)	52 (24%)	119 (53%)	< 0.001

*ACA* anterior cerebral artery, *DCI* delayed cerebral ischemia, *DM* diabetes mellitus, *HSS* Hijdra sum score, *HT* hypertension, *ICA* internal carotid artery, *MCA* middle cerebral artery, *mRS* modified Rankin Scale, *SD* standard deviation, *WFNS* world federation of neurosurgical societies

### Primary Outcomes

Univariate analysis revealed significant correlations between a poor mRS and age (OR: 1.07, *p* < 0.001), smoking history (OR: 0.57, *p* = 0.019), mFisher scale of 3–4 (OR: 1.99, *p* = 0.01), WFNS grade of Ⅳ–Ⅴ (OR: 8.05, *p* < 0.001), maximum aneurysmal size (OR: 1.12, *p* = 0.001), HSS on admission (OR: 1.09, *p* < 0.001), and poor clearance (OR: 3.51, *p* < 0.001) (Table 2). Multivariate analysis revealed significant correlations between age [odds ratio (OR), 1.03; *p* < 0.001], WFNS grade of IV–V (OR, 6.44; *p* < 0.001), and poor clearance (OR, 2.32; *p* = 0.003) (Table 2).

**Table 2:** Univariate and multivariate analysis for poor mRS, DCI, and shunt- dependent hydrocephalus

	Poor mRS				DCI				Shunt-dependent hydrocephalus			
	Univariate		Multivariate		Univariate		Multivariate		Univariate		Multivariate	
	OR (95% CI)	*P*-value	OR (95% CI)	*P*-value	OR (95% CI)	*P*-value	OR (95% CI)	*P*-value	OR (95% CI)	*P*-value	OR (95% CI)	*P*-value
Age	1.07 (1.05–1.08)	< 0.001	1.03 (1.03–1.08)	< 0.001	1.04 (1.02–1.06)	< 0.001	1.03 (1.01–1.05)	0.015	1.03 (1.01–1.04)	0.003	1.01 (0.99–1.03)	0.316
Sex, female	1.10 (0.74–1.65)	0.631			0.82 (0.51–1.33)	0.424			1.21 (0.77–1.94)	0.412		
HT	1.28 (0.86–1.90)	0.225			1.75 (1.07–2.92)	0.028	1.45 (0.85–2.51)	0.175	1.39 (0.89–2.21)	0.152		
DM	1.70 (0.85–3.39)	0.13			1.37 (0.59–2.93)	0.439			0.86 (0.36–1.88)	0.723		
Smoking	0.57 (0.36–0.91)	0.019	0.84 (0.44–1.59)	0.586	0.68 (0.38–1.18)	0.177			0.88 (0.52–1.47)	0.627		
mFisher scale 3–4	1.99 1.20–3.41)	0.01	0.67 (0.3–1.5)	0.33	3.49 (1.65–8.57)	0.003	1.80 (0.76–4.81)	0.205	3.36 (1.70–7.42)	0.001	1.44 (0.64–3.47)	0.394
WFNS grade IV–V	8.05 (5.25–12.53)	< 0.001	6.44 (3.48–12.27)	< 0.001	3.59 (2.22–5.91)	< 0.001	2.19 (1.24–3.89)	0.007	3.93 (2.50–6.27)	<0.001	2.16 (1.27–3.72)	0.005
Aneurysmal size (mm)	1.12 (1.05–1.19)	0.001	1.03 (0.95–1.12)	0.502	0.99 (0.92–1.07)	0.894			1.06 (0.99–1.13)	0.105		
HSS on admission	1.09 (1.06–1.12)	< 0.001	1.03 (0.99–1.07)	0.156	1.08 (1.05–1.11)	< 0.001	1.03 (0.99–1.07)	0.124	1.09 (1.06–1.12)	<0.001	1.05 (1.02–1.09)	0.005
Poor clearance	3.51 (2.35–5.30)	< 0.001	2.32 (1.33–4.09)	0.003	2.54 (1.56–4.21)	< 0.001	1.83 (1.09–3.14)	0.025	3.35 (2.11–5.45)	<0.001	2.55 (1.55–4.26)	< 0.001

*CI* confidence interval, *DCI* delayed cerebral ischemia, *DM* diabetes mellitus, *HSS* Hijdra sum score, *HT* hypertension, *mFisher* modified Fisher, *mRS* modified Rankin Scale, *OR* odds ratio, *WFNS* world federation of neurosurgical societies

### Secondary Outcomes

#### DCI

Univariate analysis revealed significant correlations between DCI and age [odds ratio (OR), 1.04; *p* < 0.001], HT (OR, 1.75; *p* = 0.028), modified Fisher scale of 3–4 (OR, 3.49; *p* = 0.003), WFNS grade of IV–V (OR, 3.59; *p* < 0.001), HSS on admission (OR, 1.08; *p* < 0.001), and poor clearance (OR, 2.54; *p* < 0.001) (Table 2). Multivariate analysis revealed significant correlations between age [odds ratio (OR), 1.03; *p* = 0.015], WFNS grade IV–V (OR, 2.19; *p* = 0.007), and poor clearance (OR, 1.83; *p* = 0.025) (Table 2).

### Shunt-Dependent Hydrocephalus

Univariate analysis revealed significant correlations between shunt-dependent hydrocephalus and age [odds ratio (OR), 1.03; *p* = 0.003], mFisher scale of 3–4 (OR, 3.36; *p* = 0.001), WFNS grade of IV–V (OR, 3.93; *p* < 0.001), HSS on admission (OR, 1.09; *p* < 0.001), and poor clearance (OR, 3.35; *p* < 0.001) (Table 2). Multivariate analysis revealed significant correlations between WFNS grades of IV–V (OR, 2.16; *p* = 0.005), HSS on admission (OR, 1.05; *p* = 0.005), and poor clearance (OR: 2.55, *p* < 0.001) (Table 2).

### Causal Mediation Analysis

**Fig. 2:**
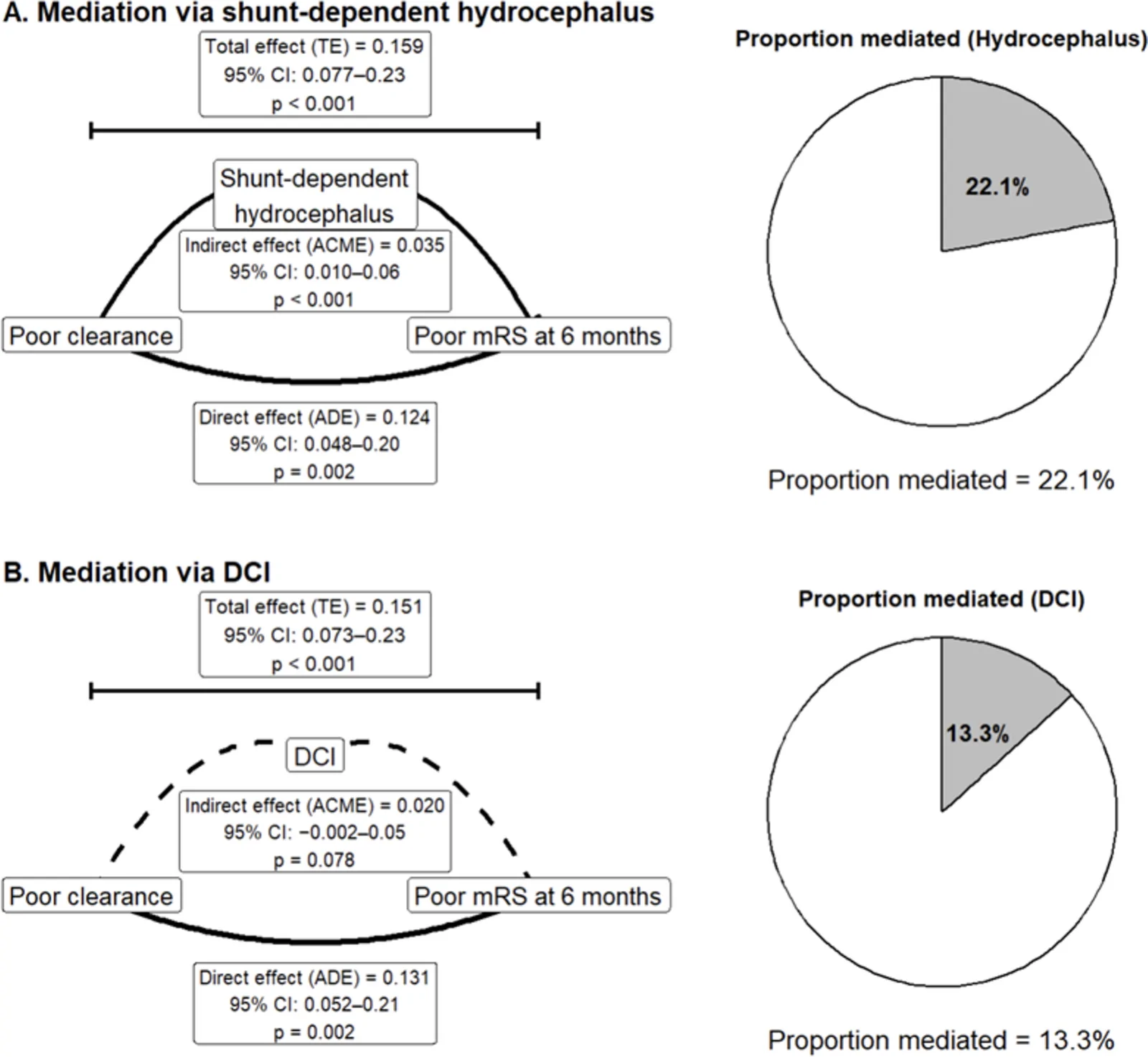
Causal mediation analyses were performed to evaluate the mediating effect of DCI in the association between HSS clearance_*Days 7–10*_ and poor outcomes at 6 months in patients with aneurysmal subarachnoid hemorrhage. ACME, average causal mediation effect; DCI, delayed cerebral ischemia; Hijdra sum score; mRS, modified Rankin Scale; TE, total effect

In the causal mediation analysis, we included the factors that are consistently included in the multivariate logistic regression analyses. These factors were: age, mFisher scale of 3–4, WFNS grade of IV–V, HSS on admission and poor clearance. Regarding the mediating effect of shunt-dependent hydrocephalus on the relationship between poor clearance and mRS, both ADE and ACME were significant (ADE = 0.124, 95% CI: 0.048–0.20, *p* = 0.002; ACME = 0.035, 95% CI: 0.010–0.06, *p* < 0.001). Additionally, shunt-dependent hydrocephalus mediated approximately 22% (proportion mediated = 0.221, 95% CI: 0.059–0.46) of the total effect (total effect = 0.159, 95% CI: 0.077–0.23). In contrast, the ACME mediated by DCI did not reach statistical significance (ACME = 0.020, 95% CI: −0.002 to 0.05, *p* = 0.078), but the ADE was significant (ADE = 0.131, 95% CI: 0.052–0.21, *p* = 0.002). Additionally, approximately 13% of the overall effect was estimated to be mediated by DCI (proportion mediated = 0.133, 95% CI: −0.019 to 0.35, *p* = 0.092) (Fig. 2).

### Robustness Analysis

The univariate and multivariate analyses using different thresholds showed similar trends for each primary and secondary outcome (Supplementary Tables 1‒3).

In the robustness analysis of the causal mediating models, the total effect remained statistically significant (25th: 0.198, *p* < 0.001; 75th: 0.142, *p* = 0.002), consistent with the main analysis. For DCI, the estimated ACME was 0.027 (*p* = 0.006) when the 25th percentile threshold was applied and 0.024 (*p* = 0.080) when the 75th percentile threshold was applied. For shunt-dependent hydrocephalus, the ACME was 0.020 (*p* = 0.068) and 0.027 (*p* = 0.018) at the 25th and 75th percentiles, respectively. In both mediators, the direction of the ACME was consistent, but the level of statistical significance varied depending on the threshold used.

### Clazosentan Comparison

There were no significant differences in the primary or secondary outcomes between the patients who were and were not administered clazosentan. The results are presented in Supplementary Table 4.

## Discussion

This study demonstrated that poor hematoma clearance in aSAH is associated with poor long-term outcomes, shunt-dependent hydrocephalus, and DCI. Furthermore, the mediation analysis revealed that the association between the poor hematoma clearance and poor long-term outcome was explained by both indirect effects mediated by shunt-dependent hydrocephalus and DCI, as well as a substantial direct effect independent of these mediating pathologies. The findings demonstrated consistent directional robustness of these associations.

To date, there have been a small number of reports on the relationship between hematoma clearance and clinical outcomes in patients with aSAH. Brouwers et al. reported that better hematoma clearance was significantly related to a better Glasgow Coma Scale score 3 months after the onset of aSAH [12]. Subsequently, Reilly et al. showed an association between hematoma clearance and cerebral vasospasm [14], and Zeineddine et al. reported that hematoma clearance was associated with DCI and mRS scores 30 days after aSAH onset [13]. Only one previous study has suggested that improved hematoma clearance may reduce the occurrence of shunt-dependent hydrocephalus [23]. The present study demonstrated an association between hematoma clearance and clinical outcomes in a larger cohort (442 patients).

Beyond the simple association, the causal mediation analysis quantified the contribution of DCI and shunt-dependent hydrocephalus to the relationship between hematoma clearance and long-term outcomes. Approximately one-fifth of the total effect was mediated through shunt-dependent hydrocephalus, and DCI accounted for a smaller proportion. Both mediators showed consistent directions of effect across different threshold definitions, suggesting that the pathophysiologic pathways through hydrocephalus and DCI are biologically plausible. The variation in statistical significance implies that the magnitude of mediation is not constant across the range of hematoma clearance.

The direct effect contributed more substantially to the relationship between hematoma clearance and long-term outcomes. The direct effect estimated in this study may include the influence of unmeasured confounders such as other mediating pathologies or differences in postoperative management, which could not be fully accounted for in the model. Other pathologies include neuroinflammation, superficial siderosis (SS), and EBI [1]. Neuroinflammation and SS potentially mediate the association between hematoma clearance and long-term outcomes. Neuroinflammation promotes neurodegeneration during the chronic phase of aSAH [24]. This pathology develops because of activation of microglia and astrocytes, resulting in the emission of cytokines such as IL-1β, TNF-α, and IL-6 [25]. SS is a condition that occurs when hemosiderin is deposited in the brain tissue after degradation of blood products [26] and is associated with cognitive impairment [27].

EBI is unlikely to mediate the relationship between hematoma clearance and the long-term outcomes of aSAH. It represents a general pathology of brain damage that occurs within 72 h of hemorrhage [28], triggered by a sudden increase in intracranial pressure and ischemia leading to blood–brain barrier disruption, microcirculatory impairment, neuroinflammation, and oxidative stress [29]. Because hematoma clearance was evaluated on days 7–10 after onset in the present study, impaired clearance cannot be considered a direct cause of EBI. However, EBI may act as a confounding factor. Severe primary brain injury may simultaneously contribute to both poor clearance and unfavorable outcomes. In our cohort, the poor clearance group more frequently exhibited WFNS grades IV–V. The higher WFNS grade is correlated with the severity of EBI [30, 31]. Therefore, although a direct causal relationship between hematoma clearance and EBI is unlikely, both variables may reflect the shared severity of brain damage. Furthermore, examination of the histogram of the reduction rate_*Days 7–10*_ reveals that, while most patients clustered around 100%, a distinct subgroup demonstrated notably poor hematoma clearance (Supplementary Fig. 1). These patients likely represent individuals with severe brain tissue injury.

Our findings may have implications for therapeutic strategies aimed at promoting hematoma clearance. In our cohort, a substantial proportion of patients underwent cisternal lavage using ventricular and cisternal drains. Recent studies have suggested that cisternal drainage may reduce secondary complications after aSAH clipping, although results remain heterogeneous [32–35]. The present study is consistent with these observations. Importantly, our data suggest that hematoma clearance is not uniform across all patients, and a distinct subgroup demonstrated markedly impaired clearance. This observation raises the possibility that clearance-oriented strategies may have differential effects depending on clot burden and individual patient characteristics. Future prospective studies are warranted to determine whether specific approaches—such as endovascular or surgical treatment combined with ventricular, spinal, or cisternal drainage, or intrathecal administration of vasodilating agents—can safely and effectively enhance clearance in selected populations.

This study has several limitations. The first is study design limitation. The present study is a single-center retrospective study; therefore, the selected population may have been subject to facility-specific biases. We excluded patients who died within 7 days because clot clearance could not be evaluated. This may introduce survivor bias, potentially underestimating the association between poor clearance and poor outcome. The second is measurement-related limitations. Clot burden was assessed using the HSS, a semi-quantitative and observer-dependent grading system. It does not provide volumetric quantification of subarachnoid blood. Therefore, measurement variability and limited precision compared with automated or volumetric imaging analyses cannot be excluded. We did not separately analyze cisternal and intraventricular components in the main mediation models. Because the biological impact of clot location may differ, this may have limited our ability to fully characterize location-specific effects of hematoma clearance. Although we defined DCI according to previous studies [18], this definition may not fully reflect the actual pathology. In this study, the definition of DCI adopted was “the presence of local neurological deficits or GCS of 2 points or more on days 4–14, or CT/MRI findings suggestive of infarction.” This may result in cases with poor baseline consciousness or neurological signs that cannot be evaluated being underestimated as well as missed cases involving microembolism, microcirculatory dysfunction, and diffuse depolarization, which are difficult to detect on imaging. Systematic electroencephalographic monitoring was not routinely performed in this cohort. Therefore, nonconvulsive status epilepticus could not be distinguished from neurological deterioration attributed to DCI. Radiological findings reflecting mass effect, such as midline shift, were not systematically quantified in this study. Because midline shift may reflect the severity of EBI and intracranial hypertension, the absence of this variable may have resulted in residual confounding. The third is treatment variability. Variations in treatment strategies, such as postoperative management and chronic rehabilitation, may be potential confounding factors. This study did not adjust for factors such as the use of antispasmodics, brain and CSF lavage, lumbar drain management, and blood pressure management during postoperative care and chronic rehabilitation. Although nimodipine is a class Ⅰ level A recommendation in the latest American Heart Association/American Stroke Association (AHA/ASA) guidelines for the treatment of patients with SAH, it is not available in Japan. Therefore, our results may not be directly generalizable to regions where nimodipine is routinely administered. The fourth is residual confounding. Although WFNS grade was included in multivariable analyses, residual confounding related to EBI severity may persist. Patients with more severe primary injury may simultaneously exhibit impaired clearance and poor long-term outcomes, which cannot be fully accounted for in a retrospective design.

## Conclusions

Poor subarachnoid hematoma clearance was associated with DCI, hydrocephalus, and poor long-term outcomes in patients with aSAH. The causal mediation analysis quantified the relative contribution of the mediating factors. DCI and hydrocephalus accounted for a modest proportion of the association between hematoma clearance and poor long-term outcomes, whereas the effect not mediated by these factors contributed more substantially to the overall relationship.

## Supplementary Information

Below is the link to the electronic supplementary material.

Supplementary file1 (DOCX 18 KB)

Supplementary file2 (DOCX 18 KB)

Supplementary file3 (DOCX 17 KB)

Supplementary file4 (DOCX 15 KB)

Supplementary file5 (PDF 571 KB)
