# Supplementary material for: Association of Impaired Hematoma Clearance After Aneurysmal Subarachnoid Hemorrhage with Delayed Cerebral Ischemia, Hydrocephalus, and Long-Term Outcomes
Source: Neurocrit Care. 2026 Apr 23;45(2):663–72. doi: 10.1007/s12028-026-02525-z (PMC13642417; doi:10.1007/s12028-026-02525-z)
Supplement: Supplementary file 1 [file 12028_2026_2525_MOESM5_ESM.pdf]

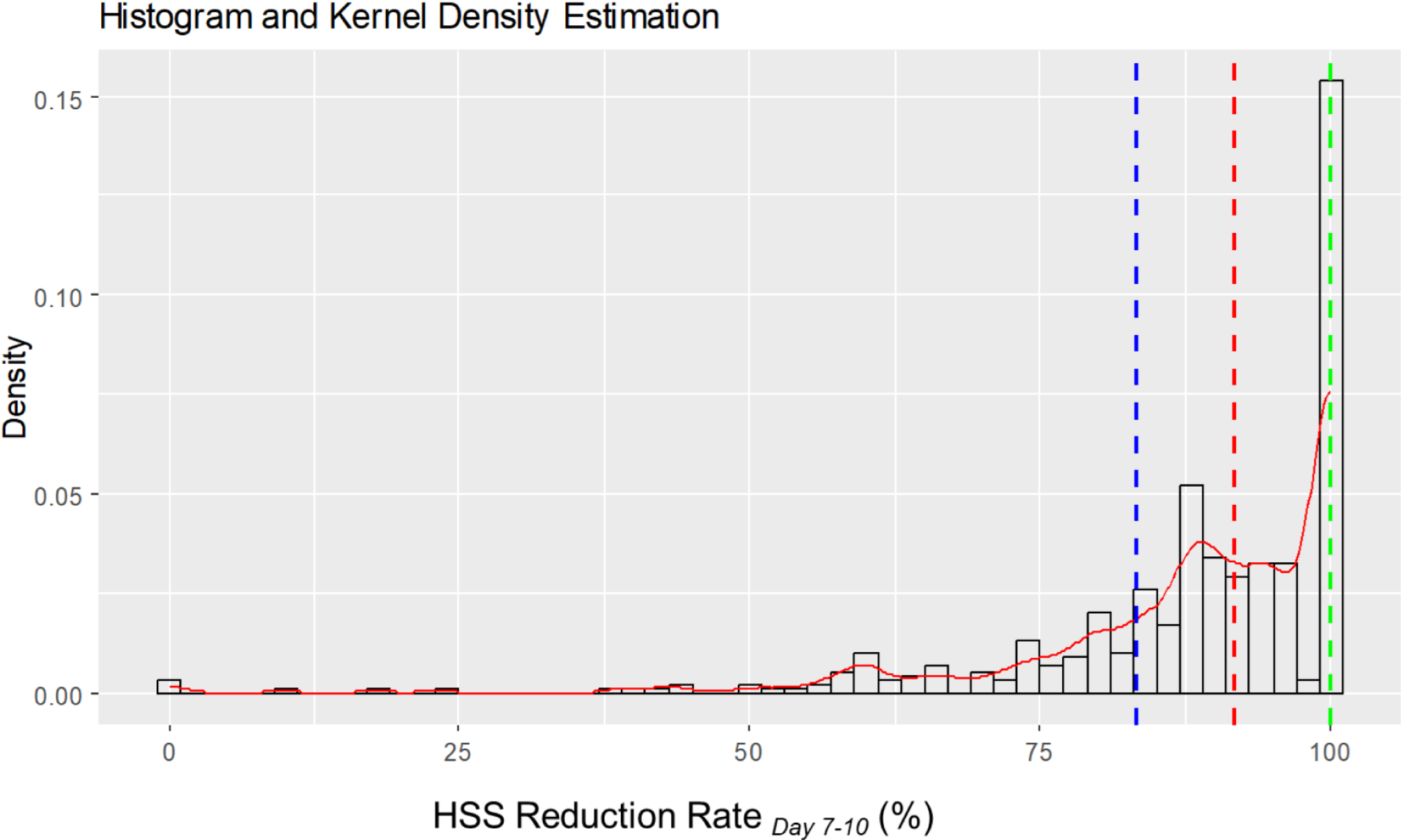
Histogram and Kernel Density Estimation
Density
0.15
0.10
0.05
0.00
0
25
50
75
100
HSS Reduction Rate Day 7-10 (%)
