# Supplementary material for: Association of Impaired Hematoma Clearance After Aneurysmal Subarachnoid Hemorrhage with Delayed Cerebral Ischemia, Hydrocephalus, and Long-Term Outcomes
Source: Neurocrit Care. 2026 Apr 23;45(2):663–72. doi: 10.1007/s12028-026-02525-z (PMC13642417; doi:10.1007/s12028-026-02525-z)
Supplement: Supplementary file 2 [file 12028_2026_2525_MOESM1_ESM.docx]

Supplementary Table 1. Assessment of the robustness for multivariate analysis of poor mRS.

|  | 25^th^ percentile | | 75^th^ percentile | |
| --- | --- | --- | --- | --- |
|  | Odds Ratio (95% CI) | p-value | Odds Ratio (95% CI) | p-value |
| Age | 1.06 (1.03 - 1.08) | <0.001 | 1.06 (1.03 - 1.08) | <0.001 |
| Smoke | 0.88 (0.48 - 1.72) | 0.774 | 0.91 (0.46 - 1.66) | 0.686 |
| mFisher group of 3–4 | 0.7 (0.31 - 1.57) | 0.388 | 0.7 (0.31 - 1.56) | 0.377 |
| WFNS grade of Ⅳ–Ⅴ | 6.42 (3.48 - 12.34) | <0.001 | 6.46 (3.47 - 12.22) | <0.001 |
| Dome size (mm) | 1.02 (0.94 - 1.11) | 0.67 | 1.02 (0.94 - 1.11) | 0.597 |
| HSS on admission | 1.02 (1 - 1.08) | 0.066 | 1.04 (0.98 - 1.06) | 0.399 |
| Poor Clearance | 2.65 (1.69 - 6.38) | <0.001 | 3.25 (1.37 - 5.33) | 0.005 |

CI, confidence interval; HSS, Hijdra sum score; mFisher, modified Fisher; mRS, modified Rankin Scale; WFNS, World Federation of Neurosurgical Societies.
