# Supplementary material for: Association of Impaired Hematoma Clearance After Aneurysmal Subarachnoid Hemorrhage with Delayed Cerebral Ischemia, Hydrocephalus, and Long-Term Outcomes
Source: Neurocrit Care. 2026 Apr 23;45(2):663–72. doi: 10.1007/s12028-026-02525-z (PMC13642417; doi:10.1007/s12028-026-02525-z)
Supplement: Supplementary file 3 [file 12028_2026_2525_MOESM2_ESM.docx]

|  | 25^th^ percentile | | 75^th^ percentile | |
| --- | --- | --- | --- | --- |
|  | Odds Ratio (95% CI) | p-value | Odds Ratio (95% CI) | p-value |
| Age | 1.02 (1 - 1.04) | 0.039 | 1.02 (1 - 1.05) | 0.018 |
| HT | 1.48 (0.86 - 2.58) | 0.159 | 1.38 (0.82 - 2.39) | 0.231 |
| mFisher group of 3–4 | 1.82 (0.76 - 4.9) | 0.20 | 1.82 (0.76 - 4.85) | 0.199 |
| WFNS grade of Ⅳ–Ⅴ | 2.1 (1.18 - 3.75) | 0.012 | 2.16 (1.23 - 3.85) | 0.008 |
| HSS on admission | 1.03 (1 - 1.07) | 0.067 | 1.02 (0.99 - 1.06) | 0.212 |
| Poor Clearance | 2.69 (1.56 - 4.64) | <0.001 | 2.05 (1.03 - 4.41) | 0.051 |

Supplementary Table 2. Assessment of the robustness for multivariate analysis for DCI.

CI, confidence interval; DCI, delayed cerebral ischemia; HSS, Hijdra sum score; HT, hypertension; WFNS, World Federation of Neurosurgical Societies.
