# Supplementary material for: Association of Impaired Hematoma Clearance After Aneurysmal Subarachnoid Hemorrhage with Delayed Cerebral Ischemia, Hydrocephalus, and Long-Term Outcomes
Source: Neurocrit Care. 2026 Apr 23;45(2):663–72. doi: 10.1007/s12028-026-02525-z (PMC13642417; doi:10.1007/s12028-026-02525-z)
Supplement: Supplementary file 4 [file 12028_2026_2525_MOESM3_ESM.docx]

|  | 25^th^ percentile | | 75^th^ percentile | |
| --- | --- | --- | --- | --- |
|  | Odds Ratio (95% CI) | p-value | Odds Ratio (95% CI) | p-value |
| Age | 1.01 (0.99 - 1.03) | 0.28 | 1.01  (0.99 - 1.03) | 0.316 |
| mFisher group of 3–4 | 1.53 (0.69 - 3.69) | 0.314 | 1.45  (0.65 - 3.5) | 0.385 |
| WFNS grade of Ⅳ–Ⅴ | 2.26 (1.34 - 3.85) | 0.002 | 2.17  (1.27 - 3.72) | 0.005 |
| HSS on admission | 1.05 (1.02 - 1.09) | 0.003 | 1.05  (1.02 - 1.09) | 0.005 |
| Poor Clearance | 1.85 (1.09 - 3.14) | 0.022 | 2.55  (1.55 - 4.25) | < 0.001 |

Supplementary Table 3. Assessment of the robustness for the multivariate analysis of shunt-dependent hydrocephalus.

CI, confidence interval; HSS, Hijdra sum score; mFisher, modified Fisher; WFNS, World Federation of Neurosurgical Societies.
