# Supplementary material for: Association of Impaired Hematoma Clearance After Aneurysmal Subarachnoid Hemorrhage with Delayed Cerebral Ischemia, Hydrocephalus, and Long-Term Outcomes
Source: Neurocrit Care. 2026 Apr 23;45(2):663–72. doi: 10.1007/s12028-026-02525-z (PMC13642417; doi:10.1007/s12028-026-02525-z)
Supplement: Supplementary file 5 [file 12028_2026_2525_MOESM4_ESM.docx]

|  | Clazosentan (Pivlaz) | |  |
| --- | --- | --- | --- |
| Outcomes | No clazosentan  N = 397 | Clazosentan  N = 45 | p-value |
| Poor outcome | 153 (38.5%) | 18 (40.0%) | 0.873 |
| DCI | 82 (20.7%) | 8 (17.8%) | 0.845 |
| Shunt-dependent hydrocephalus | 95 (23.9%) | 14 (31.1%) | 0.280 |

DCI, delayed cerebral ischemia
